# Enteric Fever

**Published:** 1861-02

**Authors:** John Richard Wardell

**Affiliations:** Editor of the M. R. C. P., Tunbridge Wells


*Enteric Fever.* By JOHN RICHARD WARDELL, M. D., Ed. M. R. C. P.

The subject of continued fever is one which in recent years has had bestowed upon it much patient and careful observation. The four varieties, viz., typhus, enteric, relapsing, and febricula, were formerly regarded under the first appellation as a generic term, and the differences which are now accounted distinct characteristics were not acknowledged. The doctrine of their identity was not doubted, and it has only been from data deduced from a very large number of cases that the questions of their etiology and pathology have been placed beyond the pale of dispute. It is very important that the views which have by so much labor been eliminated should be broadly known, because a right interpretation of the symptoms peculiar to each kind often involves weighty consequences, not only in the treatment, but in the prevention of their diffusion. Hospital physicians, and those practising in urban communities, where fevers are more rife, can at once recognise the particular species; but it must be confessed there are those in the profession whose opportunities are less ample, and who from time to time experience some difficulty touching many points associated with continued fever. With regard to the essential nature of the first three forms enumerated above, there is now no longer any doubt that they are as specifically different as are small-pox, measles, and scarlet fever. To this way of thinking I did not subscribe when, in 1846–'7, I published a series of papers in the *Medical Gazette*, founded on copious notes taken at the bedside of more than 1200 cases of fever in the hospitals at Edinburg, for the establishment of the fact of the non-identity of, and other considerations connected with, typhus and relapsing fever. I then believed the enteric (typhoid) and typhus to be the same. Dr. Jenner's able monograph proved them to be dissimilar, and, too, that the enteric and relapsing differ. The following is an example of the enteric form, condensed from the more extended report in my case-book, and I shall endeavor briefly to observe how its description talleis or disagrees with the other two named varieties:

On the 21st of June I was requested to see E. S——, a girl nine years of age, who was reported to be ill with fever. On the 12th she had had shivering and other initiatory symptoms. Her bowels not having been moved for three days, a mild aperient and an enema were ordered. The febrile expresssion was marked; in the night she had been delirious, and repeatedly endeavored to get out of bed. The eyes were suffused, the pupils dilated, and there was exceeding deafness; she was, however, quite sensible, and correctly answered questions. Lips dry; tongue moist, centre covered with a yellowish-white fur, tip and edges preternaturally red. No affection of fauces; no pain at epigastrium. Abdomen somewhat tympanitic, and gentle pressure at the iliac fossa gave the transient expression of pain. No gurgling. Auscultation discovered no signs worthy of note, nor was there any dulness over any part of the thorax. Pulse 132, weak; respiration accelerated. On a cursory review of the case, it was evidently one of enteric fever. The small, circular, rose-colored spots, acknowledged as the distinguishing diagnostic marks of that disease, could not now, after a very careful examination, nor at any subsequent date of the illness, be discovered. The head was shaved, cold applied, and wine, beef-tea, and a diapnoretic mixture were ordered.

June 23d.—A feculent stool followed the third dose of a rhubarb mixture. Tympanitis increased; pulse 130.

24th.—She tried to get out of bed during the night. Eyes suffused; pulse 138, but of larger volume; tongue moist and almost clean.

27th.—She is not so deaf; pulse 120; belly still resonant, and feels dry and harsh.

For some days afterwards the report of the case presents no facts worthy of relation. She slept better; tongue and lips were moist and clean; she was quite collected whenever I saw her; and the bowels were moved once a day, either spontaneously or by enemata. The urine from the first had been voided in normal quantity. Two small ash-colored patches appeared on the mucous membrane of the upper and under lips, which readily disappeared after being touched with a piece of alum.

On July 9th, the pulse counted the lowest to which it ever fell, 116; and I hoped she was now in a fair way of recovery, although she was by no means in the apyrexial condition.

On the morning of the 12th, there was an increase of the febrile expression; the skin was hot and dry; the abdomen more distended; pulse 134.

17th. The pulse had risen to 140, small and compressible; very deaf; tongue and lips moist and clean. Wine, during the last four days, had been increased.

18th. Much worse; lay on her back, and down in bed with knees up; considerable tympanitis; tongue moist and clean; eyes blanched, and the features had begun to sharpen. At intervals complained of pain in the abdomen. Quite sensible, and answered questions correctly. A mixture, with chloric ether, ammonia, and camphor, was prescribed, and wine and beef-tea given every hour. Pulse 144, small and thready.

19th.—Pulse 140; tongue moist; enema produced a dark feculent dejection; urine passed normally. From this time she obstinately refused to take any thing except cold water. The heart's action became more and more feeble, systolic sound brief and sharp, the face more pinched, the skin cold and clammy, until she died, in the evening of the 24th, the forty-third day after the rigors, headache and general indisposition had come on. During the last five or six days of her life there were, daily, sickness and vomiting. The pain in the abdomen was not so marked at the close as to make me suspect perforation. She sank from asthenia. There had never been the least tendency to diarrhœa, nor any loss of blood per anum. Until within a few hours of her decease she was rational; and up to the last twenty-four hours she could partially turn on the side. There was no smell of putridity, no sore or excoriation on any part of the skin, no subsultus, no picking at the bed-clothes, and the sphincters retained their functions almost to the close.

I did not press for a post-mortem examination, because it was so evident that the chief leisions would be the ordinaryly-seen ulceration of Peyer's patches, and because that morbid appearance in this form of fever has so recently been ably and in full detail described by Dr. Tweedie, who, in his clear and comprehensive Lumleian Lectures, has illustrated this pathological condition.

After a series of questions put to the mother relative to the mode of invasion, the particulars elicited were not so pathognomonic of the enteric as occurs more frequently. It begins more insidiously than typhus, and much more so than relapsing. In the latter the attack is sometimes quite sudden; a person may go to bed comparatively well, and in the morning be seized with shivering, headache, pains in the limbs, and other symptoms. There is less dulness and stupidness in the enteric than in typhus, in which the great nervous centres seem prone to be more readily impressed, as if the poison at once affected animal life. It is quite true, however, that all forms of continued fever in the symtomatology of accession present much in common, and, under an experienced eye, it is often difficult during the first few days, or even until the rash appears, to arrive at a decided diagnosis. In typhus, the muscular system evinces great prostration, not only at the first, but throughout the disease; as a rule, the patient lies helplessly on his back. In the instance given, she could turn herself in bed, and lie on the side up to a short time previous to dissolution. Again, the intellectual faculties were comparatively little disordered. During the night there was some wandering delirium, as evinced by repeated efforts to get out of bed; but in the daytime she was up to the last quite rational. The moment I entered the room she always recognised me, and, when she could be made to hear, correctly answered questions. The deafness came on early, and it varied much; some days she was very deaf, on others slightly so. This symptom is common to both typhus and enteric, and, regarded alone, it does not importantly affect the prognosis. Dr. Jenner has noticed that in the enteric type patients have a greater tendency to "get out of bed" than in typhus; the delirium is of a more vivacious kind; they are less easily restrained. In many cases the delirium does not come on until the second or third week; when it does in the first week, it is to be looked upon unfavorably. In typhus, it supervenes before the end of the first week, when it increases, and in fatal cases ends in coma. In relapsing fever, there is comparatively little delirium and head complication. On reference to my own account of that disease, in one table giving particulars of 450 cases, leeches were applied to the head, on the average, to 1 in 6.62; in another set of 80 cases it was a predominating symptom in the small proportion of 1 in 11.42. According to Louis, the brain and its membranes of those dying of the enteric rarely present any such marked appearance as might be deemed of potential consequence entering into the causation of death. To those who have had sonsiderable experience in morbid anatomy, it is well known that the encephalic mass gives little explanation of the essential nature of continued fevers. A small amount of sub-arachnoid and venticular effusion, a pinkish blush of the cortical substance, or it may be a few puncta in the centrum ovale, constitute often the entirety of morbid phenomena, and I have repeatedly examined this organ when it evinced no lesion. Even coma may not be traced after death. There is no doubt that the views enunciated by the late Dr. Clutterbuck and those professing similar opinions—regarding fever to consist of inflammation of the brain —led to much mischief in practice, and that a more correct pathology (which is, it cannot be denied, an approach to the once widely received and then discarded humoral doctrine) has been succeeded by a more successful mode of treatment —viz., that treatment which as far as possible dispenses with depletive measures, which is more expectant and conservative in its aims, and which, by having a just relation to a set of morbid actions not primarily located in any single organ or separate tissue, but pervading the entire organism, opposes asthenia. The late Dr. Alison, whose clinical assistant I was, and whose practice I carefully observed, used to dwell with much emphasis upon the Cullenian maxim of "averting the tendency to death;" and although he did not carry the wine and brandy remedy to the extreme which by some it has latterly been advocated, yet he had long seen, with much sagacity, that acute diseases, and more especially fevers, had, from some subtle and inexplicable cause, become less sthenic in their nature, demanding remedies which in previous years were accounted implicable or decidedly prejudicial.

Those organs and surfaces which are of an eliminative, a depurative, or defecating character—those emunctories and outlets whereby the products of organic waste and effete matters are carried off, are precisely those which are most liable to become affected in the progress of fever. That poison which by a most rapid multiple has so vastly increased as to contaminate the whole of the circulating fluid, in obedience to certain vital laws inherent in the organism, by such channels becomes expelled; hence, according to the power of such agent, the excess or defect of function of such organs and surfaces; and thus it is that the skin, the mucous membranes, and the parenchyma of secernent viscera more especially manifest the effects of the specific poison. Ancient theory and every-day facts convince us of those conservative qualities which the system possesses, whereby it essays to eject what it cannot assimilate, and to get rid of such noxious matter as it may have contracted. In the exanthemata, the skin and the mucous membranes of the air-passages are the chief seats of its determinations; in enteric fever, the pustulation is in the digestive tube, mainly in the ileum; in typhus, the mulberry rash evinces an effort made at the super fices to throw off the poison; and in relapsing fever, the powerful diaphoresis, which so frequently at once resolves the fever, inculcates the same doctrine. It now being conceded that the three eruptions peculiar to the respective types of continued fever, are as pathognomonic of those varieties as are the eruptions in the exanthemata, properly so called, it would really seem that it is merely an arbitrary distinction on the part of systemizers and compilers of nosology, whereby they are differently arranged. They all have many features in common; they all originate from peculiar morbid poisons, requiring a greater or less period of incubation; they all pass through a certain train of febrile phenomena; and in all the great centres of animal and organic life, are in varied degree affected. Again, there are many points of resemblance in the complications which arise, in the demand of treatment, in the sequelæ, and in the phenomena of the fatal issue. If we were to make a hasty comparison between small-pox and spotted typhus, how many characteristics they manifest of a like nature, still unquestionably caused by poisons, with which like only produces like. Both originate from peculiar poisons, are contagious, require a certain period for development, are diseases in which the prime agent specially operates upon and multiplies in the blood; in one the eruption may be looked for on the third, in the other on the sixth day; in both are all the pyrexial symptoms, as a quick circulation, high temperature, furred tongue, anorexia, diminished secretions, and a great impress is made on the nervous system; in each organic complications of the inflammatory kind may arise; there is delirium and often coma. In the one form, death mostly takes place on or about the eleventh day; in the other, rarely till after the twentieth; and both may have sequelæ attacking the same organs and tissues. And similar comparisons might be drawn between two types taken from continued fevers and the exanthems. Although human reasoning cannot say why it is that in one variety the pustulation is in the intestines, in another in the skin, yet accumulated observations have long shown that between the skin and the mucous membrane of the digestive tract, there is a peculiar sympathy, and that between the enteric and variolous phenomena many of the fundamental symptoms exemplify no slight or casual feateres of resemblance.

Dr. Kennedy, of Dublin, in a paper read a few months ago before the Medical and Chirurgical Society, propounds the doctrine that typhus and typhoid (enteric) are mere varieties originating from the same poison. Dr. Jenner's facts are opposed to this opinion. Of relapsing and typhus I can speak with much certainty. Sixteen years ago I maintained from very elaborate data their distinct essence, and such doctrine still holds good. In more than 1200 cases I never saw typhus and relapsing blended. The infection caught from one fever never produced the other. Like always produced like in a multitude of instances. I have given in my papers thirty-two cases, in which the two forms succeeded each other within a short space of time. Seventeen out of the thirty-two who had passed through the relapsing, contracted typhus during convalescence, or within the brief period of three months. The proofs of the non-identity of their essential cause were as clear as the common-sense proofs we have, and as practice ever tells us, of the non-identity of small-pox and scarlet fever. If typhus differs from the relapsing, why may it not differ from the enteric?

With regard to the moisture and cleanness of the tongue, as reported, Louis notices the same. In the 40 cases which he gives, in one-half it remained moist. In typhus, it sooner becomes brown and dry, and sordes are more common. In the relapsing, it is much furred, but moist. There was tympanitis at the first visit. This symptom is, of course, from gas in the colon; and when liquid is there also, gurgling is heard. The absence of diarrhœa accounts for the gurgling never having been recognised. In the enteric form diarrhœa is wellnigh always present. It occurred in 37 out of the 40

cases of Louis; and Drs. Tweedie, Jenner, and Wilkes regard its presence as the rule. In typhus, meteorismus and diarrhœa are rare; the belly is generally flattened or drawn in; a harass of the bowels is not spontaneous, but an incidental and seldom-observed circumstance. In the relapsing fever, in 450 cases I found diarrhœa to average 1 in 6·25; but one in 1.65, or 10 out of every 16, had urgent nausea or vomiting. When vomiting comes on in the latter part of enteric fever, ulceration of, or a changed condition in, the mucous membrane of the stomach may be suspected. According to Carpenter and other advanced physiologists, the intestinal glands are outlets for the impurities of the blood. If such be the fact, the proneness of the agminate glands to disease in the enteric form can better be accounted for. Rokitansky gives an elaborate account of the pathological changes undergone by these organs in the fever considered. He says there are four stages—one of blood stasis or congestion, a second of typhous process (infiltration of deposit,) a third of softening, and a fourth of ulceration. The lower third of the ileum, and around the cæcal valve, are the parts most affected. I remember making many examinations of the digestive tube of patients dying in fever when I was assistant in the pathological theatre in Edinburg, and such was the fact. These were then regarded as illustrations of *typhus*, the difference between it and the enteric not having then been demonstrated. I never saw ulceration in the intestines of those who died from the relapsing. So long as the typhous dyscrasia continues, thus long will the disease of these glands be maintained. The diarrhœa which ushers in the close of phthisis is accompanied by ulceration of Peyer's patches. I was much struck with the general resemblance of this patient to the appearance of those dying in phthisis. A casual observer might almost have mistaken her affection for that complaint. It is seen that the pulse never fell to the non-febrile standard. From first to last, the skin was never moist, but of dry, harsh feel, and of exalted temperature. The pulse was one day 116; it varied, seldom being exactly the same on consecutive days. Irregularity of the pulse is pathognomonic of the enteric. In typhus, when the pulse reaches 130, the case is generally fatal. In relapsing, the pulse might be exceedingly high without indicating danger. The death of E. G—— occurred at a protracted period—the forty-third day. Of twenty-five fatal cases given by Dr. Jenner of typhus, nine died on or before the fifteenth day—not one after the twenty-second. The same authority gives the twenty-second as the day mostly fatal in typhoid, (enteric,) and the fourteenth as mostly fatal in typhus. In idiopathic peritonitis the intellectual faculties keep unclouded almost to the last. The great sympathetics are potently affected, and the proximate cause of death is increasing debility of the central organs of circulation. The fatal phenomena are exerted on the action of the heart. In the enteric fever, the decrease of power in that organ demands watchful attention; the patient sinks from asthenia—hence the imperative need of stimulants.

Some writers on fever have latterly maintained, after much labor touching the etiology of continued fevers, that the enteric is always very intimately associated with some local cause. I do not, however, here enter into that vexed question as to whether malaria can or cannot of *itself* constitute a sufficient cause. I doubt that doctrine. Nor do I agree with Dr. Budd, of Clifton, that the germs of the enteric are always conveyed by the drains. I may here hastily mention that a nightman of this place informed me he had been sixteen years thus occupied, yet never had fever. Again, he added that he could instance the names of a dozen other men who for long periods had thus been employed, but he never knew one to have fever. No one, however, can gainsay the fact of bad drainage being one main element entering into the causation of fevers. The family of the deceased, whose case I have related, came to reside at Turnbridge Wells, six weeks prior to her attack. The cottage in which they live is one of a row of new, well-built houses, thoroughly ventilated, and situated in an airy, open, dry, well-drained street. The rents vary from £11 to £12, and none of the occupants can be classed with the poor. I was astonished to hear of the many cases of fever which from time to time had occurred in these houses, and felt certain of some local cause. I found that at No. 1 a family had resided five years, and four of the inmates had had fever. At No. 2 there had been six fever illnesses in nine years. At No. 3, the husband had fever some few years ago. At No. 4, where the deceased died, a young man was very dangerously ill of the same disease three years since. At No. 5, a girl had had fever, and it was reported their predecessors had it. At No. 6 I could hear of no case. At No. 7, two years ago a girl of sixteen and a boy of twelve, had "low fever;" and, within a few months, a woman of twenty-six had "typhoid fever." I was told it had often been remarked that "fresh comers" took fever. Behind the buildings are seven small strips of garden ground, and in each a privy. From the privies runs a deep drain, through a sandy shale; and within a few feet of this drain is the pump-well. The well-water had long been complained of, and, after heavy rains, it was said to have a bad taste. The town surveyor, who made an inspection, told me he believed it very probable that the matter from the drains might percolate into the well. He recommended the well to be filled up. In the next set of premises is another well, supplying two other houses. I made inquiries but could not learn of any inmate ever having fever in these two dwellings; but they always used the water from their own pump, and never from the pump common to the seven houses. The chief cause of the diffusion of typhus is destitution; for proof of which I would refer the reader to the records of Scotch and Irish epidemics. In relapsing fever, of 436 cases, 81 were in partial work, and 301 were destitute. The relapsing fever cases at Edinburg and other large towns in Scotland, were in a less relative proportion in the ground-floor houses, whose occupyers were mostly little traders, and in comparatively better circumstances, though, of course, nearer the drains than those above them; but in the upper flats there was by far more poverty, and, consequently, far more fever.—*London Lancet.*

*Tunbridge Wells*, 1860.

